# Expression of Concern: Protective effect of autophagy on endoplasmic reticulum stress-induced apoptosis of alveolar epithelial cells in rat models of COPD

**DOI:** 10.1042/BSR-20170803_EOC

**Published:** 2020-07-27

**Keywords:** Alveolar epithelial cells, Apoptosis, Autophagy, Chronic obstructive pulmonary disease, Endoplasmic reticulum stress

The Editorial Office has been made aware of potential issues surrounding the scientific validity of this paper, hence has issued an expression of concern to notify readers whilst the Editorial Office investigates.

